# Supplementary material for: Real-World Analysis of Nivolumab and Atezolizumab Efficacy in Previously Treated Patients with Advanced Non-Small Cell Lung Cancer
Source: Pharmaceuticals (Basel). 2022 Apr 25;15(5):533. doi: 10.3390/ph15050533 (PMC9147485; doi:10.3390/ph15050533)
Supplement: Supplementary file 1 [file pharmaceuticals-15-00533-s001.zip › pharmaceuticals-1661657-supplementary.pdf]

Supplementary Appendix.

Table S1. Baseline Characteristics by histological group.

Characteristic	All patients (n=158)	Squamous (n=71)	Non-squamous (n=87)	p-value
Age, median (range), years	64 (37-86)	67 (45-81)	61 (37-86)	<0.001‡
Binary age at ICI begining				<0.001*
≤65	90 (57)	29 (40.8)	61 (70.1)	
>65	68 (43)	42 (59.2)	26 (29.9)	
Sex				0.202*
Male	124 (78.5)	59 (83.1)	65 (74.7)	
Female	34 (21.5)	12 (16.9)	22 (25.3)	
ECOG at ICI initiation				0.215*
0-1	134 (84.8)	63 (88.7)	71 (81.6)	
≥2	24 (15.2)	8 (11.3)	16 (18.4	
Metastasis CNS	26 (16.5)	5 (7)	21 (24.1)	0.004*
COPD	45 (28.8)	26 (36.6)	19 (21.8)	0.041*
PD-L1 (positive ≥1% tumor cells)				0.107*
Yes	60 (38.0)	26 (88.7)	34 (39.1)	
No	37 (23.4)	12 (16.9)	25 (28.7)	
Unknown	61 (38.6)	33 (46.5)	28 (32.2)	
Smoking status				0.298*
Never or +10 years former smokers	42 (26.6)	16 (22.5)	26 (29.9)	
Current or -10 years former smokers	116 (73.4)	55 (77.5)	61 (70.1)	

‡ p value for t student test, * p value for Chi-squared test; ECOG, Eastern Cooperative Oncology Group; CNS, central nervous system; COPD, Chronic Obstructive Pulmonary Disease

Table S2. Univariate analysis of baseline characteristics that were significantly different between nivolumab and atezolizumab groups.

		Nivolumab						Atezolizumab					
		PFS			OS			PFS			OS		
Parameter	Category	HR	95% CI	p value	HR	95% CI	p value	HR	95% CI	p value	HR	95% CI	p value
Age	≤65 vs. >65	1.08	0.68-1.70	0.742	1.03	0.63-1.69	0.901	1.25	0.69-2.24	0.456	0.94	0.50-1.79	0.860
CNS met	No vs. Yes	1.09	0.54-2.20	0.803	1.52	0.65-3.52	0.331	0.73	0.40-1.35	0.320	0.89	0.45-1.77	0.749
Histology	Sq vs. non-sq	1.58	0.96-2.60	0.072	1.54	0.91-2.63	0.108	1.21	0.64-2.26	0.556	1.93	1.00-3.72	0.050
PD-L1	- vs. +	1.43	0.68-3.02	0.341	0.99	0.42-2.32	0.986	1.20	0.67-2.14	0.536	1.17	0.61-2.22	0.640
	Unk vs. +	1.29	0.75-2.22	0.351	1.22	0.68-2.19	0.502	0.45	0.19-1.05	0.066	0.52	0.22-1.25	0.145

CNS met, central nervous system metastasis; PFS, progression free survival; OS, overall survival; HR, hazard ratio; CI, confidence interval.
